# Micro-RNAs -106a and -362-3p in Peripheral Blood of Inflammatory Bowel Disease Patients

**DOI:** 10.2174/1874091X01812010078

**Published:** 2018-06-29

**Authors:** Ameneh Omidbakhsh, Mohsen Saeedi, Masoud Khoshnia, Abdoljalal Marjani, Safoura Hakimi

**Affiliations:** 1 Student Research Committee, Gorgan Faculty of Medicine, Golestan University of Medical Sciences, Gorgan, Golestan province, Iran; 2 Stem Cell Research Center, Gorgan Faculty of Medicine, Golestan University of Medical Sciences, Gorgan, Golestan province, Iran; 3 Golestan Research Center of Gastroenterology and Hepatology, Gorgan Faculty of Medicine, Golestan University of Medical Sciences, Gorgan, Golestan province, Iran; 4 Metabolic Disorders Research Center, Department of Biochemistry and Biophysics, Faculty of Medicine, Golestan University of Medical Sciences, Gorgan, Golestan province, Iran

**Keywords:** MicroRNA-106a, MicroRNA-362-3p, Inflammatory Bowel Disease, Biomarkers, Crohn's Disease, Ulcerative Colitis

## Abstract

**Objective:**

MicroRNAs (miRNAs) can regulate various genes after binding to target mRNAs. Studies on Inflammatory Bowel Disease (IBD) in relation with miRNA are much less shown. The aim of the present study was to assess the expression patterns of microRNA 106a and microRNA 362-3p in peripheral blood samples of Inflammatory Bowel Disease (IBD) patients including Crohn’s Disease(CD) and Ulcerative Colitis (UC).

**Methods:**

This study consisted of 32 CD, 32 UC patients and 32 controls. The expression level of the micro-RNAs -106a and -362-3p was determined using reverse transcription and real-time RT-PCR.

**Results:**

Our findings showed that MiR-106a and miR-362-3p are expressed at significantly higher levels in the peripheral blood from patients with CD and UC compared to controls. MiR-106a and miR-362-3p expression are also different in the peripheral blood of patients regarding the activity score of the disease. There were significant differences of miR362-3p in active UC relative to inactive UC.

**Conclusion:**

Altogether our findings suggest that miR-106a and miR-363-3p can play an important role in the pathogenesis of IBD. The differences in expression of miR106a and miR362-3p in peripheral blood of the UC and CD patients in an active phase in comparison to inactive disease suggest that these miRNAs may be useful as potential biomarkers for diagnosis and monitoring the disease activity.

## 1 INTRODUCTION

Inflammatory Bowel Disease (IBD) including Crohn’s Disease(CD) and Ulcerative Colitis (UC), the two main forms of IBD, with shared pathological and clinical symptoms. The cause of IBD is not exactly clear. Some studies have shown that genetically susceptible subject with an abnormal immune response against the microorganisms of the intestinal flora is responsible for the disease [1, 2]. Studies have indicated that there are a relationship between the pathogenesis of IBD and the adaptive immune response. It has been recently reported that the innate immune response is important as in gut inflammation [3]. The incidence of IBD is increasing [4] in the world and at very young age [5, 6] and it has attempted studies to describe the pathogenic mechanisms. The genetic mechanism accounting for the development and progression of the IBD is not exactly clear. The molecular pathology of the CD and UC indicates different, genetic and immunologic characteristics. This means that CD and UC are different kinds of disease with overlapping clinical characteristics [7-9]. Studies on expression of mRNA and proteomics have indicated that CD and UC change in several aspects [10, 11] but they also show obviously different features [12]. Genome-Wide Association Studies (GWAS) identified about 200 loci associated with the development of IBD [13-20]. This explains for 16–23% of the heritability of IBD [21-23]. Some studies have revealed that there are many other factors that influence the development of IBD such as epigenetics, epistasis, and environmental factors [24-26]. Following the release of the IBD genome studies results, researchers have been interested to explore the relationship between IBD and the microRNAs (miRNAs), as one of the main gene expression regulators. Beside the developments in modern medicine, unfortunately in many cases The IBD diagnosis happens months or years after the beginning of the clinical symptoms. For diagnose of the IBD, it is necessary to acquire different tests of clinical, radiological, endoscopic and histological experiments [27]. According to Genome-Wide Association Studies (GWAS) results have concluded to the understanding of disease etiology and the genetic arrangement, and genetic variants cause low diagnostic value for IBD [28] and some other diseases [29]. Measurements of microRNAs (miRNAs) in tissue [30] or peripheral blood of IBD patients have been shown in the context of their function in IBD [31]. Recent study by Chen *et al*. [32] has revealed microRNAs capability to serve as diagnostic markers. miRNA expression levels are more stable in tissues and peripheral blood. MicroRNAs are small RNA molecules with 20–25 nucleotides. They can regulate various genes after binding to target miRNAs. They can control the stability and translation of protein-coding mRNAs [33]. Recent studies indicated that miRNAs are expressed in UC playing important role as negative regulators of inflammation and innate immunity [34]. Most of the studies are concentrated on the role of miRNA in the development of cancer, and autoimmune diseases. Studies on IBD in relation with miRNA are much less shown. Dys-regulation of the intestinal miRNA may cause impaired barrier function and inflammation similar to IBD [35]. There are important challenges in miRNA research. Many of the miRNA with mild effects may regulate a specific gene. It is recognized that miRNAs play important roles in the cell differentiation and organogenesis. Abnormalities in miRNA expression may cause disease development [36]. It is indicated that almost one third of human genes are targets for miRNA regulation [37]. Many of miRNAs have been recognized, but there is less data about their function [38]. Study on the intestinal tract has shown that miRNAs are involved in tissue homeostasis, intestinal cell differentiation, and the maintenance of intestinal barrier function [35]. Finding of microRNAs in 1933 has indicated that they involved in multiple pathophysiological networks [39, 40] and in the pathogenesis of cancer and inflammation. There is more attention for microRNAs research recently, especially for critical roles of microRNA in IBD that microRNAs may have influence in the pathogenesis of chronic inflammation and oncogenic transformation [41-43, 33]. In the present study, the aim of study was to assess the expression patterns of microRNA 106a and microRNA 362-3p in Crohn’s Disease(CD) and Ulcerative Colitis (UC) in peripheral blood samples of Inflammatory Bowel Disease (IBD) patients.

## 2 METHODS

This study included 32 patients with CD, 32 patients with UC and 32 healthy controls. The diagnosis of either CD or UC was confirmed by gastroenterologist and standard clinical, endoscopic, radiological, histological criteria, and colonoscopy findings. The Ethical Committee of Research Deputy of Golestan University of Medical Sciences was approved the study (IR.GOUMS.REC.1394.45). Whole blood samples were used to extract total RNA by TRIzol reagent (Invitrogen,carlsbad, CA) according to manufacturer's instructions. The RNA samples were stored at −80°C. DNase I was used to treat total RNA by using Invitrogen kit. Extracted RNA quality and quantity were determined by Nanodrop. The expression patterns of the target miRNA and a housekeeping gene, U6sn, were quantitatively determined using reverse transcription and real-time RT-PCR. U6 levels were almost the same in CD and UC patients. Looped reverse transcription primers specific for each miRNA were used to synthesize Stem-loop complementary DNAs (cDNAs). Relative real-time PCR syber green method using Pars Genome commercial kit (Iran) was used to amplifying the miRNAs. A 20 μl reaction mixture was prepared for the Relative Real time-PCR process including Cyber Green master mix (10μl), cDNA (Diluted, 7-8 μl), Mix of miRNA specific primers (1 each 10 pmol). The Relative Real time-PCR amplification conditions included 1 denaturation cycle at 95°C for 30 seconds and 40 cycles of amplification at 95°C for 5 seconds, 60-63°C for 20 seconds, 72°C for 30 seconds. Specific primer for each miRNA (miScript primer assay) was provided from Pars Genome Company. We tested the expression of miR-106a, miR-362-3p. All samples were tested two times to confirm reproducibility. miR-106a, miR-362-3p and U6sn were amplified in all samples. Specific melting temperature was seen in amplified miRNAs. This confirms the exactness and specificity of the used method. Real-time-PCR was conducted on an ABI Prism 7300 apparatus (Applied Biosystems, USA). The spss-22 software was used to analyze our data. The expression of each miRNA was normalized to U6sn RNA internal control. The levels of miRNAs expression were normalized after subtracting the Ct value of the U6sn RNA internal control from that of each miRNA Ct value for samples (ΔCt=|CtmiRNA (samples)−CtU6sn|). The ΔΔCt value was determined by using the formula [ΔΔCt=ΔCtmiRNA (sample A)−ΔCtmiRNA (sample B)] for comparison of the levels of miRNA expression between the samples .The relative level of miRNA in IBD samples was compared to normal samples by setting the miRNA expression in normal samples value to 1 and determining the fold change in expression against this value using the following formula 2^-ΔΔct^. Comparison of miRNA level differences between patients and controls were done by Mann-Whitney U test. P-value of less than 0.05 was considered as statistically significant.

## 3 RESULTS

In this study, we have shown the expression of two micro-RNAs, miR-106a and miR-362-3p, in patients with ulcerative colitis, Crohn's disease and the control group. The peripheral blood of patients with active Crohn's Disease (CD), Active Ulcerative Colitis (UC), and control samples was used to extract miRNAs. The demographic characteristics and clinical features of patients with CD, UC and controls were summarized in Table **1**. 68.75% and 62.50% of inflammatory bowel disease patients had Crohn's disease and Ulcerative colitis, respectively. The rest of patients were Quiescent. There were significant differences of miR-106a and miR-362-3p expression in individual peripheral blood between groups. Regarding the CD and UC cases, miR-106a and miR-362-3p were expressed at significantly higher levels in the peripheral blood from patients with CD and UC compared to controls (Except for miR-362-3p in UC). The expression levels were shown in Table **2**. MiR-106a and miR-362-3p expression are also changed in the peripheral blood of patients with either active or inactive UC and CD. Active levels of miR-106a and miR-362-3p were higher than from those of inactive levels. These miRNAs are increasingly expressed when compared to the controls. There were no significant differences of miR-106a in active UC compared to the controls.

There were no significant differences of miR-106a and miR362-3p in active CD relative to inactive CD (Table **3**).

## 4 DISCUSSION

Many people suffer from different diseases which are related to dysregulation of the intestinal immune system and various autoimmune diseases, and cancers [44-46]. One of the most studied classes of intestinal disease is Inflammatory Bowel Disease (IBD). Some studies have revealed that miRNA expression changes in individuals suffering from IBD. The differences in expression of peripheral blood miRNAs and their important role in IBD have not been exactly studied. UC and CD Patients have various miRNA profiles which are different when compared with healthy controls [47-50]. In this study, we compared miR106a and miR362-3p expression of peripheral blood samples from patients with inflammatory bowel disease (Crohn’s disease and ulcerative colitis) compared to controls. There were an altered expression of miR106a and miR362-3p among UC and CD patients. We have also indicated that miR106a and miR362-3p distinguish patients with active IBD from those with inactive disease and from control subjects. Wu *et al*. [51] reported the differences in expression of miRNA in the mucosa of patients with active UC tissues compared to healthy tissues. They also have shown that miRNA expression was different in healthy controls compared to quiescent UC patients. Wu *et al*. [52] also indicated that ileum and sigmoid colon tissues prepared from CD patients expressed distinct miRNAs. Studies have shown that many kinds of miRNAs have a role in the regulation of inflammatory bowel disease. There are limited studies on the role of peripheral blood miR106a and miR362-3p in IBD. Studies of Wu *et al*. [48] and Zahm *et al*. [53] on IBD in an adult and a pediatric population have revealed that peripheral blood miRNAs can distinguish active IBD subtypes form each other and healthy controls. Studies of Wu *et al*. and Fasseu *et al*. on peripheral blood samples and sigmoid colon biopsies from active UC and CD patients have shown that miR106a and miR362-3p are increased in both patients when compared to control groups. They confirmed that miRs-362-3p increased significantly in expression [48, 54] which is in agreement with our study. Another study by Wu *et al*. indicated that mi-RNA expression is changed in biopsies of patients with active CD and ileal CD (Crohn's ileitis) and colonic CD (Crohn's colitis) [52]. Paraskevi and colleagues revealed that miR-106a and miR-362-3p were upregulated in peripheral blood from active CD patients when compared to healthy controls in IBD [55]. Studies of Zahm and colleagues on serum in place of the peripheral blood of active pediatric CD patients have indicated that miR-106a expression was significantly increased compared with healthy controls [53]. In the present study, using real-time PCR, we determined that miRNAs significantly increased in both CD and UC blood samples compared to controls. Our results are consistent with some other findings of miRNAs expression in IBD blood samples [48, 53-55]. We have indicated that peripheral blood miR106a and miR362-3p are present in both CD and UC.

MiR-106a and miR-362-3p in active CD patients exhibited the largest increase in expression. This means that these miRNAs may be used to separate these two diseases. Recent study has been shown detection of differential miRNA expression in blood samples [47]. It has been reported that CD and UC differ in tissue miRNA and peripheral blood miRNA Profiles [47]. Some studies have revealed that miR-362-3p was increased in the peripheral blood of patients with active and inactive CD and UC [47] compared to controls which is in agreement with our results. Wu *et al*. also identified and validated the increased expression of miR-362-3p in peripheral blood samples from both UC and CD patients [48]. Study of Taganov *et al*. [56] indicated that there are no differences between the miRNA expression among the patients with active CD and Crohn's ileitis and Crohn's colitis sub-groups. This means that there are no miRNAs common expression between the tissue biopsies and the blood samples in patients with Crohn's colitis, Crohn's ileitis and active UC [56]. Determination of peripheral blood miRNA expression in IBD patients relative to healthy controls revealed elevated expression of miR-362-3p in Crohn’s disease and ulcerative colitis [48-50]. Findings of Duttagupta*et al* [57]. revealed that miRNA expression in peripheral blood miR-106a and miR-362-3p elevated in Crohn’s disease and ulcerative colitis. These studies were inconsistent with our findings. Recent studies have suggested that circulating miRNAs may play a role as biomarkers in CD patients. Zahm *et al*. [53] showed that miRNAs differentially expressed in the serum of patients with CD compared with healthy controls. MicroRNA, mir-106a could differentiate UC from CD in biopsy samples and increased in Crohn’scolitis and not in UC [52]. A study demonstrated that CD and UC differ not only in their tissue miRNA profiles but also in their peripheral blood miRNA profiles [48]. Some other studies have shown that miR-362-3p increased in active CD and not in inactive CD in the blood of patients [52] which is inconsistent with our findings. Elevated expression of these miRNAs in CD and UC diseases suggests that these miRNAs may play an important role in inflammatory disorders. The determination of molecular mechanism and the diagnoses of ulcerative colitis and Crohns disease remain unclear. The overexpression of miR-106a in Crohns disease, not in ulcerative colitis may be associated with the particular clinical presentation and clinical outcome of Crohns disease. There were some limitations in our study. miRNAs expression may be affected by medical treatment of some patients. However, both of miRNAs expression in active CD and UC were higher than inactive CD and UC compared to controls. Thus, these miRNAs could distinguish active and inactive CD and UC, and controls from each other. This may help to predict and understand CD and UC pathogenesis.

## CONCLUSION

In conclusion, our findings indicated that miR106a and miR362-3p are overexpressed in peripheral blood of patients indicating an important role in the pathogenesis of IBD. The differences in expression of miR106a and miR362-3p in UC and CD blood samples with active or inactive disease suggest that these miRNAs may be useful as potential biomarkers for diagnosis or monitoring tool in future.

## Figures and Tables

**Table 1:** Demographic characteristics and clinical features of patients with Crohn's Disease (CD), Ulcerative Colitis (UC) and controls.

**Parameters**	**Crohn's disease (CD)** **(n = 32)**	**Ulcerative colitis (UC)** **(n = 32)**	Control (n = 32)
**Age (years)**	**32.45±11.3**	**31.53±12.28**	33.47±10.92
**Sex(men/women) (n)**	**17/15**	**17/15**	17/15
**Disease behavior:** **Inflammatory n (%)** **Quiescent n (%)**	**22(68.75)** **10(31.25)**	**20(62.50)** **12(37.50)**	-

**Table 2:** miR106a and miR362-3p expression in patients with Crohn's Disease (CD), Ulcerative Colitis (UC) compared to controls.

**Parameters**	**Fold Change of MiR106a** **(Mean ± SD)**	**P. Value**	**Fold Change of MiR362-3p** **(Mean ± SD)**	**P. Value**
**Control (n=32)**	**1**		**1**	**-**
**Ulcerative Colitis (n=32)**	**1.48±1.17**	**<0.001**	**1.59±1.54**	**0.358**
**Crohn's** **Disease (n=32)**	**3.82±5.54**	**<0.001**	**2.5±3.62**	**<0.001**

Fold change is relative to controls and shown as mean ± SD, P<0.05.

**Table 3:** Active and inactive miR106a and miR362-3p expression and their relative in patients with Crohn's Disease (CD), Ulcerative Colitis (UC) compared to controls.

**Parameters**	**Fold Change of MiR106a** **(Mean ± SD)**	**P-Value**	**Fold Change of MiR362-3p** **(Mean±SD)**	**P-Value**
**Control (n=32)**	**1**		**1**	**-**
**Active Ulcerative Colitis (n=20)**	**1.42±1.23**	**<0.001**	**1.4±1.47**	**0.492**
**Inactive Ulcerative Colitis (n=12)**	**1.2±0.56**	**<0.001**	**1.39±1.03**	**0.01**
**Active crohn's** **Disease (n=22)**	**3.08±4.82**	**<0.001**	**2.39±3.80**	**0.021**
**Inactive crohn's Disease (n=10)**	**2.61±4.60**	**<0.001**	**1.51±1.48**	**0.04**

Fold change is active disease relative to inactive disease and shown as mean±SD, P<0.05.
